# Creating the Evidence through Comparative Effectiveness Research for Interprofessional Education and Collaborative Practice by Deploying a National Intervention Network and a National Data Repository

**DOI:** 10.3390/healthcare3010146

**Published:** 2015-03-18

**Authors:** Judith Pechacek, Frank Cerra, Barbara Brandt, May Nawal Lutfiyya, Connie Delaney

**Affiliations:** National Center for Interprofessional Practice and Education, University of Minnesota, Minneapolis, MN 55455, USA; E-Mails: pech0004@umn.edu (J.P.); cerra001@umn.edu (F.C.); brandt@umn.edu (B.B.); delaney@umn.edu (C.D.)

**Keywords:** National Center for Interprofessional Practice and Education, Nexus incubator network, NCDR, intervention research, interprofessional education, collaborative practice, Nexus of Inquiry, comparative effectiveness research

## Abstract

*Background*: There is currently a resurgence of interest in interprofessional education and collaborative practice (IPECP) and its potential to positively impact health outcomes at both the patient level and population level, healthcare delivery, and health professions education. This resurgence of interest led to the creation of the National Center on Interprofessional Collaborative Practice and Education in October 2012. *Methods*: This paper describes three intertwined knowledge generation strategies of the National Center on Interprofessional Practice and Education: (1) the development of a Nexus Incubator Network, (2) the undertaking of comparative effectiveness research, and (3) the creation of a National Center Data Repository. *Results*: As these strategies are implemented over time they will result in the production of empirically grounded knowledge regarding the direction and scope of the impact, if any, of IPECP on well-defined health and healthcare outcomes including the possible improvement of the patient experience of care. *Conclusions*: Among the motivating factors for the National Center and the three strategies adopted and addressed herein is the need for rigorously produced, scientifically sound evidence regarding IPECP and whether or not it has the capacity to positively affect the patient experience of care, the health of populations, and the per capita cost of healthcare.

## 1. Background

In October 2012, based on a national competitive process, a public-private partnership comprised of the Health Resources and Services Administration (HRSA), the Josiah Macy Jr. Foundation, the Robert Wood Johnson Foundation (RWJF), and the Gordon and Betty Moore Foundation funded the National Center for Interprofessional Practice and Education at the University of Minnesota (hereafter the National Center) [1]. The creation of the National Center, in large measure, grew out of a resurgence of interest in interprofessional education and collaborative practice (IPECP) and the potential ability of IPECP to positively impact health outcomes, healthcare delivery, and health professions education [2]. Even though there is global interest in IPECP, current healthcare reform efforts embodied by the Affordable Care Act [3] in the United States (US) and the innovation already occurring in the redesign of the processes of care delivery provided an umbrella context for the work undertaken by the National Center. The development of interprofessional practice competencies by the Interprofessional Education Collaborative (IPEC) [2] also contributed to the context supporting the significance of the National Center.

Among other things, the National Center has committed to being an unbiased source of information on the impact, if any, of IPECP on the outcomes of the Triple Aim [4]. The National Center is charged with providing an infrastructure for national interprofessional research and evaluation activities including data analysis and dissemination as described in a 2013 paper by Chen, *et al.* [5] Studying the impact of patient-centered, interprofessional team-based care on Triple Aim outcomes is the work the public-private partnership of funders are supporting. While the work of the National Center may on the surface appear to overlap with the commitments of other endeavors, such as the Patient-Centered Outcome Research Network (PCORn) funded by the Patient-Centered Outcomes Research Institute (PCORI) a full year after the National Center was established, the charges of the two endeavors are different. As such, the infrastructures of each of these efforts are really not redundant. This is true of other federally funded research related networks. Whenever possible, the National Center coordinates with other organizations and networks with overlapping or complementary interests. In light of its charge the National Center has selected three intertwined strategies to collect data, test IPECP models, and rigorously generate evidence to contribute to knowledge about IPECP impact.

The National Center has adopted the following definitions of interprofessional education and collaborative practice. At present these are the most widely accepted definitions of each.

- *Interprofessional education* “occurs when two or more professions learn about, from, and with each other to enable effective collaboration and (to) improve health outcomes.” [6].
- *Interprofessional Collaborative Practice* “…happens when multiple health-related workers from different professional backgrounds work together with patients, families, care givers and communities to deliver the highest quality of care.” [7].

A number of Cochrane reviews of randomized controlled trials (RCT) [8,9,10] have called for “…better evidence of the impact of interprofessional education on professional practice and healthcare outcomes.” [8]. Other reviews favoring observational studies [11] have also called for the production of better evidence regarding IPECP and its capacity to affect health and education outcomes. To provide unbiased information on the impact of IPECP and at the same time bolster the evidence base about IPECP, the National Center has created a *Nexus* where interprofessional education and collaborative practice intersect to inform change at the micro, meso and macro levels (Table 1), thus, contributing to redesigning healthcare delivery and health professions education [12]. The ultimate goal of the Nexus is to create a unified system between currently disparate ones (health professions education and healthcare delivery) that focus on IPECP interventions intent on achieving the Triple Aim outcomes of improving the patient experience of care, improving the health of populations, and reducing the per capita cost of care [4].

**Table 1:** Level of change defined by context.

Change Level	Clinical	Education	Nexus
Micro	Provides care of patients and operates within its own environment and ecology; participants are committed to working together	Teaching environment to education learners	Intentionally create relationship at the practice and education microsystem level to achieve the Triple Aim
Meso	Senior leadership and governing structures in clinical systems; corporate offices, governing boards	University/college presidents, provosts, deans and senior administration; governing boards and trustees, regents	Greater understanding of synergies between health system transformation and meeting higher education needs; support IPECP implementation at micro level
Macro	Political, financial, accreditation and policy environment; state, regional and/or national level; increasingly complex	Political, financial, accreditation and policy environment; state, regional and/or national level; increasingly complex	Political, financial, accreditation and policy environment; state, regional and/or national level; increasingly complex

As an integral dimension of this Nexus, the National Center is partnering with a number of what are referred to as *incubator* sites or living laboratories that have developed and are implementing IPECP interventions aimed at testing and examining how these interventions impact healthcare practice and health and interprofessional education outcomes. Further, to ensure that the relevant data being generated and collected will be available for scientifically sound analyses and knowledge creation, the National Center has developed and is currently populating the National Center Data Repository (NCDR). The NCDR has been designed to house incubator intervention data, as well as institutional-level aggregated or aggregatable data from multiple organizations on multiple domains (e.g., education, finance, ecological factors), that will eventually facilitate analyses to produce scientifically sound evidence about IPECP.

The purpose of this paper is to describe the conceptualization of the living laboratories or incubators of the National Center and to characterize the NCDR as these efforts are connected to the National Center’s commitment to provide unbiased and scientifically sound evidence on IPECP. Underpinning the incubator strategy and that of the NCDR is a commitment to a comparative effectiveness research (CER) strategy. The remainder of this paper discusses these three strategies: the incubators, the implementation of CER in this context, and the NCDR.

## 2. Methods: The Research Strategies of the National Center

### 2.1. The Incubator Strategy

The National Center has conducted and published a scoping review of IPECP literature, published between 2008 and 2013, analyzed through the lens of the Triple Aim [11]. This literature review found a paucity of research connecting IPECP to healthcare reform outcomes such as those outlined in the Triple Aim [4]. This paucity underscores a paradox—that the force driving the resurgence of interest in IPECP has a thin evidence base on which to rely to make the case for the importance of IPECP to healthcare and health professions education reform.

To begin to close this knowledge gap, the National Center has chosen to identify, select, nurture, and provide scientific and technical support to incubators that have developed and are implementing IPECP interventions aimed at impacting health and healthcare outcomes. An essential component of each of these intervention projects is conceptualized outcomes that are tied to those of the Triple Aim: the reduction of per capita healthcare costs, improvement in population health, and/or positively impacting the patient experience of healthcare [4]. Hence, a significant dimension of these incubator intervention projects is the *elevation* (from micro only level) of identified outcomes for study or assessment, data generation and analysis. The National Center by nurturing a number of incubators is engaged in *researching and assessing* the impact of well-designed and scientifically rigorous IPECP interventions. At present there are 20 incubator projects (taking place in 11 states) with plans to increase that number to at least 65 projects by 2017 (if not sooner). Table 2 summarizes the current incubator projects by intervention type, intervention team composition and outcomes being measured. Every intervention type addresses patient experience of care as well as improved health outcomes for patients as a result of team-based health care provision.

This incubator strategy is grounded in the belief that interprofessional education (IPE) is a means to an end just as collaborative practice (IPC) is [10,13,14]. IPE is an intervention that may take many different forms that ostensibly create transformative learning [14] resulting in IPC. IPC is the means, with other factors and variables, by which outcomes related to patient healthcare cost, healthcare quality, and eventual improvement in population health will be impacted [4]. The Nexus brings both of these components together in a dynamic relationship wherein both learn with, from, and about each other and focus on improving healthcare and health professions education outcomes, a relinking that produces health professionals who are prepared to practice in new models of the process of care and create a continuous improvement platform that changes as the redesign of healthcare continues to occur. Along the way, the healthcare workforce will also become better informed.

Moreover, intervention research was chosen as a knowledge creation strategy because the redesign of healthcare delivery is occurring rapidly and the need for evidence generated from real-life circumstances is imperative. This approach of defining achievable outcomes and managing to them, stimulated by the quality and safety improvement movement, is now widely adopted in the healthcare marketplace relying on experiential data that many healthcare systems are collecting and analyzing. Interventions exist on a continuum from simple to complex and wherever an intervention rests on this continuum, it is implemented as a purposive change strategy using an interprofessional team thought to be the one most likely to achieve the stated results of the intervention. [15] Each intervention has an appropriate control. While these team-based interventions are implemented at the point of care, they are also able to direct or facilitate change at many levels [12,15].

**Table 2:** Nexus incubator interventions by team composition and measured outcomes.

Project Intervention Type	Team Composition	Outcomes Measured
*Transitions of Care:* Inpatient and outpatient quality and safety Acute stroke to rehabilitation to home	*Staff:* physicians, nurse practitioners, pharmacists, physician assistants, dentists, social workers, physical therapists, occupational therapists, speech therapists, nutritionists, behavioral health, patients and family members	*Clinical:* error reduction, reduced upstream hospital readmission, reduced resource use, improved patient satisfaction with care, improved staff satisfaction with care provision, cost reductions
	*Students:* medical, advanced practice nursing, pharmacy, behavioral health, allied health, social work	*Educational:* changes in knowledge and skills, IPECP * team competencies, meaningful participation in care process
*Disease Related:* Improved outcomes in adults with diabetes Improved outcomes in patients with asthma	*Staff:* physicians, nurse practitioners, pharmacists, behavior health, dentistry, social work, patient family, administrators	*Clinical:* improved disease markers, reduced cost of care, reduced resource use, reduced disease complications, improved clinic efficiency, improved team function
	*Students:* medical, advanced practice nursing, pharmacy, behavioral health, dentistry, social work, physician assistants	*Educational:* IPECP * skills, assessment of readiness to practice
*Primary Care:* Team function to improve patient outcomes Establishing sustainable academic-clinical partnerships for IPECP *	*Staff:* physicians, nurse practitioners, pharmacists, behavior health, medical assistants, social work, clinic coordinators, clinic administrators, patients and family members, academic faculty, community partners	*Clinical:* improved patient satisfaction with care, improved staff satisfaction with care provision, improved process of efficiency, improved care coordination, reduced cost of care, reduced emergency department use, reduced use of inpatient services
	*Students:* medical, pharmacy, behavior health, social work, medical technicians, dental	*Education*: assessing team care in a learning environment, complex health care experience, assessment of team competencies and readiness for practice
*Education Institution Process Improvement:* Assessing student training sites for IPECP * readiness Faculty training preceptors for IPECP * at the point of care	*Staff:* physicians, nurse practitioners, pharmacy, dentistry	*Clinical:* TeamSTEPPS as a team training tool for hospital staff, additional knowledge and skills needed in curriculum, more effective collaborative care, improved patient satisfaction with care, improved staff satisfaction with care provision
	*Students:* medical, advanced practice nursing, pharmacy, physical therapy, occupational therapy	*Education:* TeamSTEPPS as a team training tool for students, assessing meaningful roles for students in team care, assessment tool validation, improved IPECP * learning environment, improved role modeling, behavior change, knowledge and skill acquisition
*Expanding Roles of Advanced Practice Providers (APN, PA):* In trauma rehabilitation As hospitalists	*Staff:* nurse practitioners, physician assistants, allied health, care coordinators, patients and family members, physicians as needed	*Clinical:* team performance, patient/provider satisfaction, care quality, cost and resource use reductions, comparative assessment of model performance
	*Students:* advanced practice nursing, physician assistant, social work, physical therapy, occupational therapy, dentistry	*Education:* Assessing value added and meaningful use of students on clinical care provision teams, identification of additional skills and knowledge needed, assessment of team transfer skills

* Interprofessional Education and Collaborative Practice.

Intervention research is the “…systematic study of purposive change strategies.” [15]. When people act differently because of an intervening mechanism, then concerted, directed change happens.

**Figure 1:**
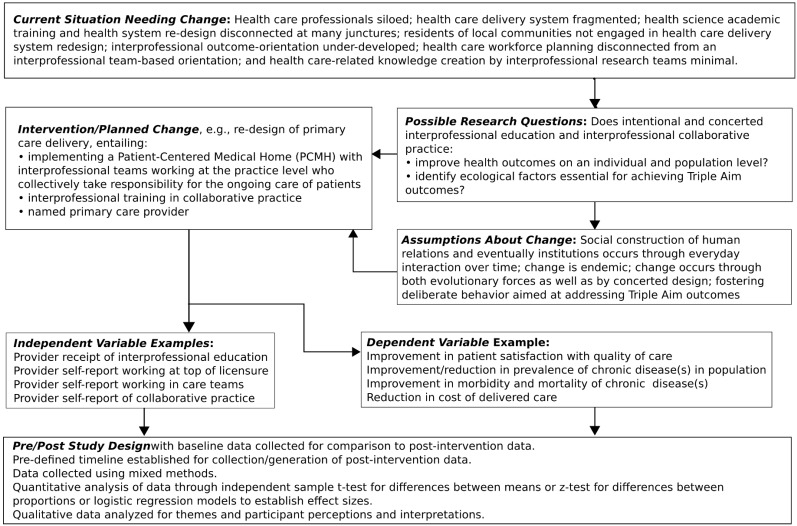
Illustration of intervention design and research process.

Interprofessional education interventions of all types, as well as interprofessional collaborative practice interventions, are examples of intervening mechanisms, as is the IPECP Nexus. The logic of interventions is that as they are implemented they will influence behavior change in those exposed and may result in subsequent changes that impact micro, meso, or macro levels (Table 1). Figure 1 illustrates the intervention research process.

We make a number of assumptions regarding why and how an IPECP intervention might impact or effect an identified dependent variable. In terms of designed change making the Triple Aim flesh, the following are among the many possible underlying assumptions:

1. A collaborative team approach to care could reduce healthcare cost.
2. Collaborative training could result in better healthcare quality by developing interdependencies, mutual respect and understanding for scope of work among health professionals.
3. Health professionals trained in understanding population health could enhance primary prevention ultimately leading to reduced prevalence of (at the very least) modifiable risk factors that may result in chronic disease. This can happen at the point of care one person at a time, a disease-based population intervention, or a population health status intervention.
4. A collaborative team approach to care could facilitate cost effectiveness with appropriate level providers working at the top of their licenses to provide care at the appropriate level of prevention (primary, secondary, or tertiary).
5. Integrating community members (patients and families) into healthcare delivery planning could enhance engagement in personal health, leading to reduced chronic disease and improved population health.

**Figure 2:**
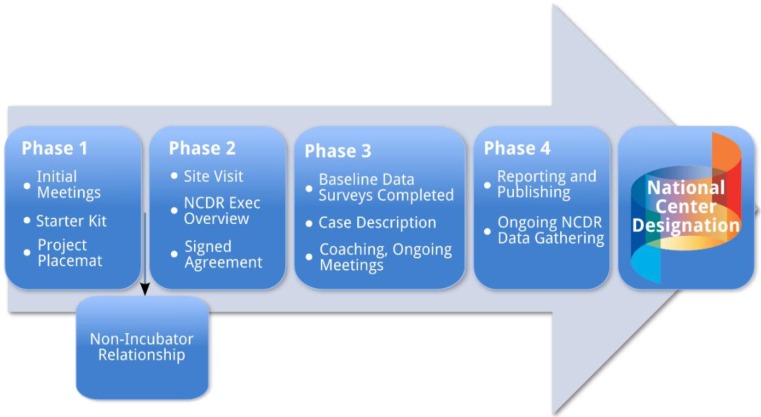
Developmental phases of nexus incubator projects.

Generalizable findings that also account for the environmental-ecological factors that affect the achievement of the desired outcomes are vital if the hope of developing a sound evidentiary base of the impact of IPECP Nexi on health outcomes is to be realized. For findings to be generalizable they must be generated from rigorously designed intervention research with sufficient sample sizes to allow for analyses that can produce and assess the effect sizes of predictor variables on outcome variables. Hence the National Center has developed and implemented a phased approach to selecting intervention projects to be part of its Nexus Incubator Network. Each phase involves a set of obligations on behalf of both the National Center and the incubators. As incubator projects are implemented they receive technical support from National Center experts in the areas of research, informatics, and evaluation. This developmental, phased approach ensures that well-developed, scientifically sound interventions are implemented and assessed. The phases are summarized in Figure 2.

### 2.2. The CER Strategy

The Affordable Care Act [3] explicitly connected comparative effectiveness research (CER) with current healthcare reform in the US. This is not without controversy since randomized control trials (RCT) alone are often thought of as the gold standard for advancing scientific knowledge creation [16,17,18]. RCTs are a form of intervention research, [15] and is one among many study designs classified as CER [19,20,21]. Whilst the gold-standard in the current hierarchy of evidence is that produced by RCT, this is much too limiting when interventions are outside the scope of specific types of clinical interventions such as drug or device studies [18].

Often RCTs produce findings with limited generalizability based on inadequate contextual information from small samples [18,19]. Evidence produced from RCTs relies on a very narrow and specific definition of causality where the timing and controlling of events leads to the inference that one variable led to and even created another [19,20]. Other criteria for scientific evidence may be equally or more valuable for informing practice and policy [19]. Well-designed observational studies or quasi-experimental interventions (such as the ones undertaken by the Nexus incubators) frequently produce valuable evidence that can and should inform action [19,20,21,22]. Others [23,24,25] have made a similar case suggesting that what they refer to as the 5Rs is a *bold* standard for conducting relevant research in a changing world [23]. Proponents of the 5Rs offer that the changing paradigm of healthcare research needs to (1) be relevant to stakeholders, (2) be rapid and recursive, (3) redefine rigor while maintaining scientific integrity, (4) report on required resources needed to implement interventions, and (5) be replicable [23,24,25].

Some questions are better or even best answered by longitudinal studies or studies that are not as controlled as RCTs. For example, what is the impact of IPECP interventions on sustainable change in the process of care; the scalability/transportability of a new model of care; or the effects on population health? In such instances it makes sense to create opportunities to connect the dots from the findings from a *number* of well-designed studies in order to build a relevant, reliable and valid knowledge or evidence base employing both qualitative and quantitative data. Connecting the dots entails linking evidence from research on specific steps in a *likely* causal pathway by creating a pathway of linked association. [21,22] Inferences made to linked knowledge between associations should be supported by well-developed logic models and clearly articulated theories of change (plausible explanations for change) [21,22].

The concept of a pathway of linked associations was initially introduced by Braveman [21,22] to address the question of *when we know enough to recommend action* on a health-related question or concept. Similar to Braveman *et al.* [22], Phelan *et al.* [26] have suggested that the potential fundamental root causes of health and/or health related outcomes (e.g., in our case interprofessional education and/or collaborative practice) can be identified using a similar connect the dots logic based on the following four essential elements: the influence on multiple disease or health outcomes; impact on health outcomes through multiple risk factors; impacting access to resources that can be used to avoid risks or minimize the consequences of disease; and whether or not a relationship is reproduced over time through the replacement of intervening mechanisms. Connecting evidence from different studies addressing these elements allows for the identification of plausible critical components of a pathway leading to a health-related outcome.

**Figure 3:**
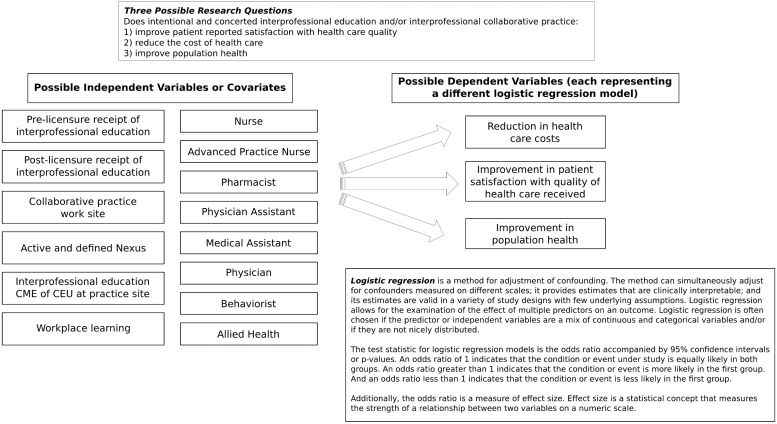
Example of logistic regression analysis models for examining impact of IPECP on triple aim outcomes.

In the instance of IPECP within the current context of the US healthcare system reform, data generated for decision making requires a CER approach relying on an informatics platform for the generation and subsequent use of large scale databases that are amenable to both cross sectional and longitudinal analyses. Figure 3 displays a possible analytic approach. The data collected need to capture sentinel events that when analyzed can produce timely and actionable information to facilitate making the best decisions possible in a rapidly changing healthcare environment. In practice, outcome goals and metrics are established and interventions are implemented and modified on an ongoing basis to quickly move toward desired outcomes.

### 2.3. The National Center Data Repository (NCDR) Strategy

The third strategy to be discussed is that related to the creation of a relational database to provide analyzable data to examine the impact of IPECP on the patient experience of care, the cost of health care provided, and the health of populations. The National Center has developed and is currently populating a relational database, the NCDR, to collect and store, not only incubator project specific data, but also data from entities across the nation involved in IPECP initiatives. The former will allow for the analysis of data generated from intervention focused work while the latter will facilitate the generation of big data for observational studies. The NCDR platform also provides a *sandbox* for the development, validation and deployment of new and/or improved assessment tools regarding interprofessional teams. As it is populated, the NCDR will provide a sound informatics foundation for the generation of new knowledge regarding the impact of IPECP over time. The NCDR was designed to address the decades-old challenge (mentioned earlier) of the lack of data to test and assess the effectiveness of interprofessional models in achieving improved outcomes.

The NCDR resides in the University of Minnesota Academic Health Center Information Exchange (AHC IE) and is based on a robust information architecture platform. The University of Minnesota AHC IE has existing policies and procedures in place to manage privacy, access to and governance of data [27]. Additionally, an NCDR national advisory committee, comprised of recognized experts in the field representing practice, education and informatics, has been created to advise National Center staff on issues related to implementation, metrics, and evaluation of the NCDR.

The NCDR architecture has several components, including:

**Input:** incubator project designees enter their data into a secure *user-defined* data environment with a *user-friendly* web interface.

**Core Data Set:** consisting of education content and process, cost inputs, outputs, outcomes and ecological information (for example, cultural and sociological facts about the site). A designee from each incubator project submits its National Center Core Data Set at the commencement of its innovation project and updates it annually. In some instances, data are submitted more frequently as determined by the National Center. A sentinel event reporting function is also present for use when there is any significant change at the intervention site, e.g., new payment system, new scope of practice legislation, change in leadership in C-Suite, *etc*.

**Project Data:** each incubator project creates its own project-specific database and infrastructure to document its unique IPECP interventions and outcomes. This project specific information is de-identified and captured by the NCDR. If the intervention has disease-specific outcomes (e.g., A1c levels, blood pressure, BMI), those are part of the project data. To every extent possible, the intervention site’s electronic health record (EHR) will be used as a data source. Once the project-specific intervention is completed, the outcomes will continue to be followed to assess the sustainability of change and the continued improvements of the new Nexus (e.g., process of care and aligned education). Other information systems will also be used, depending on the data required (e.g., site-specific financial systems data).

The model for the NCDR was extensively adapted from one conceptualized, although not implemented, by the Canadian Institute on Governance because of the lack of funding [28]. The National Center has studied the model and its assumptions, determining its value to inform data collection in the US. A number of significant adaptations were introduced into this Canadian return on investment (ROI) model, such as the US focus on healthcare reform grounded in the Triple Aim [4].

## 3. Projected Results: The Deliverables of the Nexus Incubator Network and the NCDR

**Figure 4:**
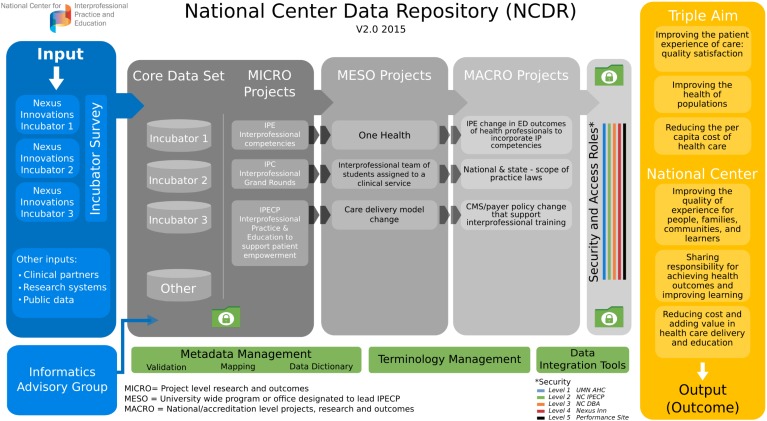
The incubator network and the national center data repository.

The interface between the incubator projects and the NCDR is dynamic and depicted in Figure 4. The incubator-database functions as a unit and is overseen by a user group, a technical group, and the National Center Management group that consists of a project manager and project coordinators. These ensure a continuous improvement process that assesses project and NCDR status, data movement, technical challenges, robustness of the aggregated data, need for sites to perform interventions of a specific type to attain robustness of a dependent variable, validation of data entered in all surveys, and the analytics that are performed on both the individual project and NCDR aggregated data.

As the robustness of the data increases, analysis will report, and update as needed, information (qualitative) and evidence (quantitative) answering the following core queries regarding whether or not IPECP will:

- Improve the Health Outcomes (Triple Aim) on an individual and population level?
- Result in improvement in educational outcomes?
- Identify ecological and/or environmental factors essential for or barriers to achieving Triple Aim health outcomes?
- Identify factors essential for or barriers to sustaining the transformation of the process of care?
- Identify changes needed in policy, accreditation, credentialing and licensing?

Establish the associations and when possible causal connections between Triple Aim health outcomes, interprofessional education and collaborative practice?

## 4. Discussion

This paper has focused on describing three intertwined knowledge generation strategies of the National Center on Interprofessional Practice and Education—the development of a Nexus Incubator Network, the undertaking of comparative effectiveness research, and the creation of a National Center Data Repository. These strategies, as they are implemented over time, will result in the production of empirically grounded knowledge regarding the direction and scope of the impact, if any, of IPECP on well-defined health and healthcare outcomes. Among the motivating factors for not only the National Center itself but also the three strategies adopted and addressed here is the need for rigorously produced and scientifically sound evidence regarding IPECP.

In the US, healthcare reform initiated by the Affordable Care Act [3], has accelerated the pressure [23] for implementable knowledge generated by, among other efforts, intervention research to answer questions about specific actions that will improve healthcare provision (safety, quality), for specific groups or populations, that will be cost effective [23]. While answering these questions is essential, quickly influencing practice (at multiple levels) is even more essential [23,24,25].

This concern with quickly moving significant research findings into practice is one focus of not only CER but also of other articulated endeavors such as 5R research (which is an example of CER) [25,26]. One recent editorial [24] has connected the dots between 5R research as an approach that could advance the Triple Aim. The knowledge creation strategies of the National Center are aligned with the efforts of 5R research, recognizing the importance of quick practice implementation whenever possible.

## 5. Conclusions

In the US, as well as globally, both interprofessional education and collaborative practice are experiencing a resurgence of interest. This interest is grounded in multiple contextual and ecological factors and these have been discussed throughout the paper. Among other things the National Center is committed to being an unbiased source of information on the impact of IPECP on patient experience of care, the cost of delivered care, and population health. To this end the National Center has chosen, developed, and is at present implementing three interrelated knowledge generation strategies described in-depth in this article.

## References

[1] National Center for Interprofessional Practice and Education. http://nexusipe.org/about.

[2] Cerra F., Brandt B. (2011). Renewed focus in the United States links interprofessional education with redesigning healthcare. J. Interprof. Care.

[3] The Patient Protection and Affordable Care Act Detailed Summary. http://www.dpc.senate.gov/healthreformbill/healthbill04.pdf.

[4] Berwick D.M., Nolan T.W., Whittington J. (2008). The triple aim: Care, health, and cost. Health Aff..

[5] Chen F.M., Williams S.D., Gardner D.B. (2013). The case for a National Center for Interprofessional Practice and Education. J. Interprof. Care.

[6] World Health Organization Department of Human Resources for Health (2010). Framework for action on interprofessional education and collaborative practice.

[7] Barr H., Waterton S. (1996). Interprofessional Education in Health and Social Care in the United Kingdom: Report of a CAIPE Survey.

[8] Zwarenstein M., Goldman J., Reeves S. (2009). Interprofessional collaboration: Effects of practice-based interventions on professional practice and healthcare outcomes. Cochrane Database Syst. Rev..

[9] Reeves S., Zwarenstein M., Goldman J., Barr H., Freeth D., Hammick M., Koppel I. (2008). Interprofessional education: Effects on professional practice and healthcare outcomes. Cochrane Database Syst. Rev..

[10] Reeves S., Perrier L., Goldman J., Freeth D., Zwarenstein M. (2013). Interprofessional education: Effects on professional practice and healthcare outcomes (update). Cochrane Database Syst. Rev..

[11] Brandt B.F., Lutfiyya M.N., King J.A., Chioreso C. (2014). A scoping review of interprofessional collaborative practice and education using the lens of the Triple Aim. J. Interprof. Care.

[12] D’Amour D., Oandasan I. (2005). Interprofessionality as the field of interprofessional practice and interprofessional education: An emerging concept. J. Interprof. Care.

[13] Hall P., Weaver L. (2001). Interdisciplinary education and teamwork: A long and winding road. Med. Educ..

[14] Frenk J., Chen L., Bhutta Z., Cohen J., Crisp N., Evans T., Fineberg H.V., Garcia P., Ke Y., Kelley P. (2010). Health professionals for a new century: Transforming education to strengthen health systems in an interdependent world. Lancet.

[15] Fraser M.W., Galinsky M.J. (2010). Steps in intervention research: Designing and developing social programs. Res. Soc. Work Pract..

[16] Moore L., Moore G. (2011). Public health evaluation: Which designs work, for whom and under what circumstances?. J. Epidemiol. Community Health.

[17] Craig P., Dieppe P., Macintyre S., Michie S., Nazareth I., Petticrew M. (2008). Developing and evaluating complex interventions: The new Medical Research Council guidance. BMJ.

[18] Safford M.M. (2014). Comparative effectiveness research and outcomes of diabetes treatment. JAMA.

[19] Dreyer N.A., Tunis S.R., Berger M., Ollendorf D., Mattox P., Gliklich R. (2010). Why observational studies should be among the tools used in comparative effectiveness research. Health Aff..

[20] Sox H.C. (2010). Defining comparative effectiveness research: The importance of getting it right. Med. Care.

[21] Bravman P.A. (2003). Monitoring equity in health and healthcare: A conceptual framework. J. Health Popul. Nutr..

[22] Braveman P.A., Egerter S.A., Woolf S.H., Marks J.S. (2011). When do we know enough to recommend action on the social determinants of health?. Am. J. Prev. Med..

[23] Peek C.J., Glasgow R.E., Stange K.C., Klesges L.M., Purcell E.P., Kessler R.S. (2014). The 5 R’s: An emerging bold standard for conducting relevant research in a changing world. Ann. Fam. Med..

[24] Ewigman B. (2014). Could 5R research help achieve the Triple Aim?. Ann. Fam. Med..

[25] Peek C.J., Cohen D.J., de Gruy F.V. (2014). Research and evaluation in the transformation of primary care. Am. Psychol..

[26] Phelan J.C., Link B.G., Tehranifar P. (2010). Social conditions as fundamental causes of health inequities: Theory, evidence, and policy implications. J. Health Soc. Behav..

[27] University of Minnesota Academic Health Center Information Exchange AHCIE Program Update. http://www.ahc.umn.edu/prod/groups/ahc/@pub/@ahc/documents/content/ahc_content_262677.pdf.

[28] Nason E. (2012). Return on Investment and a Research Agenda for Interprofessional Education and Collaborative Practice: Overview Document.

